# Supplementary material for: Nanocrystalline ZnSnN2 Prepared by Reactive Sputtering, Its Schottky Diodes and Heterojunction Solar Cells
Source: Nanomaterials (Basel). 2022 Dec 30;13(1):178. doi: 10.3390/nano13010178 (PMC9823707; doi:10.3390/nano13010178)
Supplement: Supplementary file 1 [file nanomaterials-13-00178-s001.zip › nanomaterials-2063820-supplementary.pdf]

nanomaterials

Supplementary Materials

Nanocrystalline $ZnSnN_2$ Prepared by Reactive Sputtering, Its Schottky Diodes and Heterojunction Solar Cells

Fan Ye *, Rui-Tuo Hong †, Yi-Bin Qiu †, Yi-Zhu Xie, Dong-Ping Zhang, Ping Fan and Xing-Min Cai *

Key Laboratory of Optoelectronic Devices and Systems of Ministry of Education and Guangdong Province, and Shenzhen Key Laboratory of Advanced Thin Films and Applications, College of Physics and Optoelectronic Engineering, Shenzhen University, Shenzhen 518060, China
* Correspondence: yefan@szu.edu.cn (F.Y.); caixm@szu.edu.cn (X.-M.C.)
† These authors contribute equally to this work.

The steps to prepare the Schottky diodes with the structure of Ag\ITO\$ZnSnN_2$\Ag and the solar cells (Ag\ITO\$Cu_2O$\$ZnSnN_2$\Ag and Ag\ITO\$Cu_2O$\$ZnSnN_2$\Au) are shown in **Figure S1** (ITO refers to indium tin oxide).

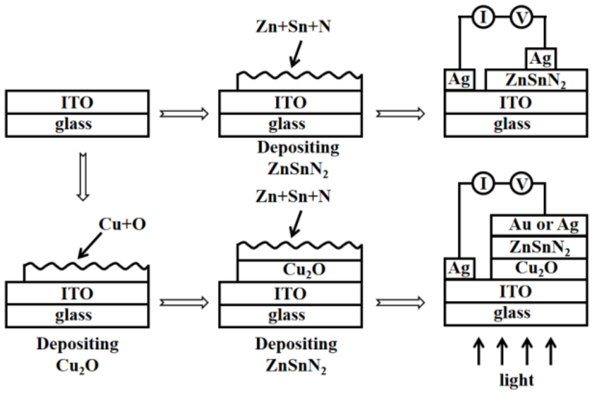

Figure S1. The steps to prepare the Schottky diodes and the heterojunction solar cells. The upper three panels are about the Schottky diodes and the lower three are about the heterojunction solar cells.

To see how the substrate temperature will affect the structure of the deposited film, $ZnSnN_2$ were deposited at different substrate temperatures but the other parameters are exactly the same with those mentioned in the manuscript. **Figure S2** shows the X-ray diffraction patterns of the samples deposited at different substrate temperatures (Room temperature to 300 ^{0}C). No diffraction peaks are observed when the substrate temperature is below 100 ^{0}C. However, diffraction peaks are observed when the substrate temperature is 100-300 ^{0}C. $ZnSnN_2$ deposited at 100-300 ^{0}C are hexagonal [1].

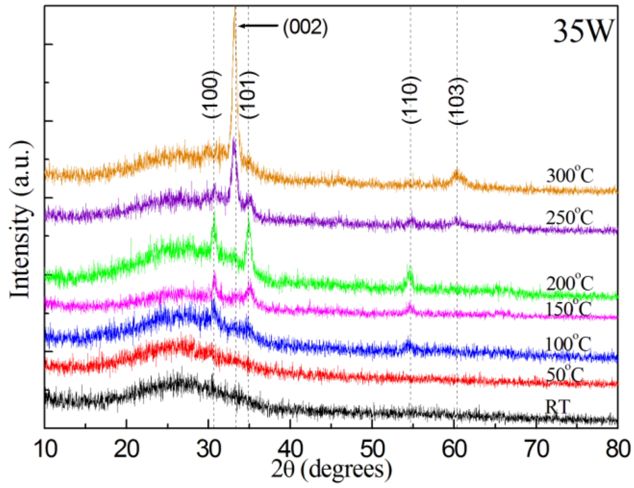

Figure S2. XRD patterns of $ZnSnN_2$ samples deposited at different substrate temperatures.

To see how the sputtering power will affect the structure of the deposited film, the sputtering power was increased to 50W and the substrate temperature of room temperature, 50, 100 and 150 ⁰C were used while other parameters were the same with those in the manuscript. **Figure S3** shows the XRD patterns of the samples prepared at 50W with the substrate temperature to be in the range of room temperature to 150 ⁰C. From **Figure S3**, it can be found that when the sputtering power was 50W, even the samples deposited at room temperature is polycrystalline [1].

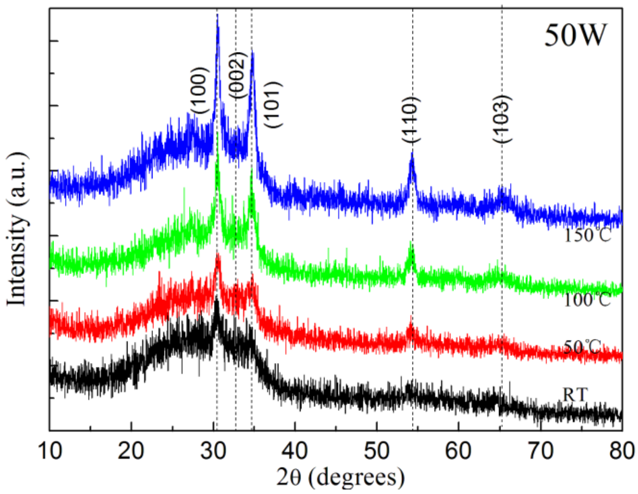

Figure S3. The XRD patterns of the samples prepared at 50W and substrate temperature is from room temperature to 150 ⁰C.

The Raman scattering spectra of the samples deposited at 35W and room temperature without annealing or annealed at 300-450 ⁰C are shown in **Figure S4**. No peaks but humps are observed in the Raman scattering spectra of these samples and this is the same with its polycrystalline counterparts [2-3]. Before annealing, humps centered at about 121, 512, 629 and 689 cm^{-1} were observed. After annealing, humps centered at 234 cm^{-1} appear in some samples and humps centered at 689 cm^{-1} disappear in all the annealed samples. These spectra are similar to the calculated high resolution phonon density of states spectrum of $ZnSnN_2$[4]. Just like crystalline or polycrystalline $ZnSnN_2$, nanocrystalline $ZnSnN_2$ is also phonon-glass-like and the k-selection rule breaks down.

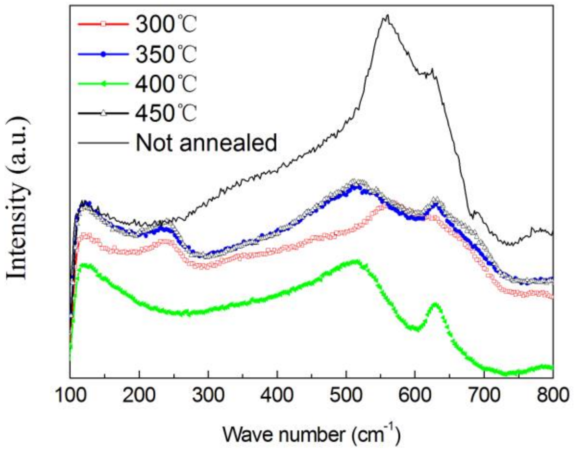

Figure S4. Raman scattering spectra of $ZnSnN_2$ samples without annealing or annealed at 300, 350, 400 and 450 ^{0}C.

Figure S5 shows the current I-voltage V curves of the structure of Ag\ITO\$ZnSnN_2$\ITO\Ag. The curves are linear for $ZnSnN_2$ without annealing or annealed at 300 ^{0}C. The samples annealed at 300 ^{0}C is found to have smaller resistivity, in agreement with the results obtained with Hall effect measurements. This implies that the rectification or nonlinearity of Ag\ITO\$ZnSnN_2$\Ag is due to the contact between $ZnSnN_2$ and Ag.

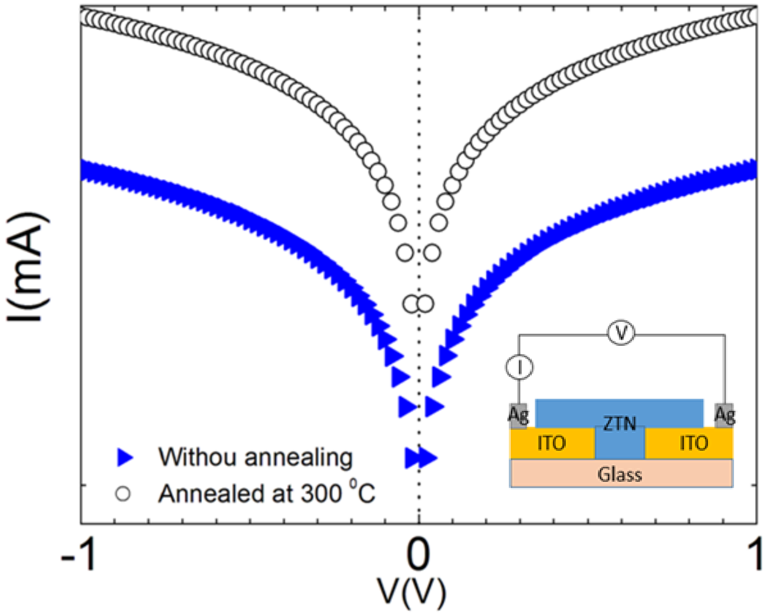

Figure S5. The IV curves of Ag/ITO/$ZnSnN_2$/ITO/Ag (the inset shows the cross-sectional view of the multilayer structure.).

The ideal band diagram of Ag-$ZnSnN_2$ at zero bias is shown in **Figure S6 (a)**. The band diagram of Ag-$ZnSnN_2$ with interface oxide at zero bias is shown in **Figure S6 (b)** [5].

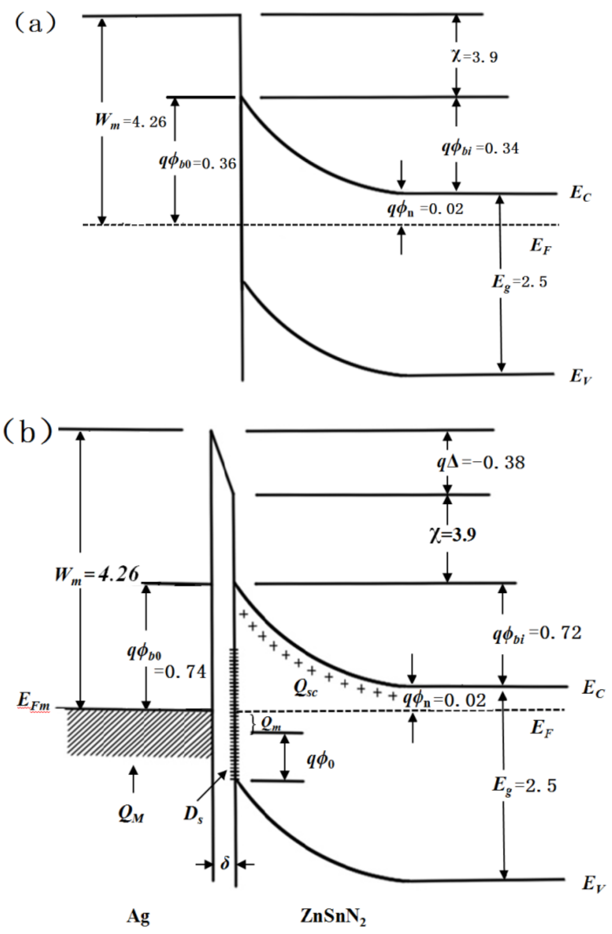

Figure S6. (a) The ideal band diagram of Ag-ZnSnN2; (b) The band diagram of Ag-$ZnSnN_2$ with interface states [5].

Figure S7 shows the statistical distribution of the performance factors of the two types of solar cells. In the horizontal axis, "Au" and "Ag" refer to the data are from the samples with Au and Ag as the electrodes without sample storage, while "Stored" refers to the data are from the samples (Ag\ITO\$Cu_2O$\$ZnSnN_2$\Au solar cell) stored in air without encapsulation for about 11 months. The performance factors include V_{oc} (open circuit voltage), J_{sc} (short-circuit current), FF (fill factor) and PCE(power conversion efficiency). For the Ag\ITO\$Cu_2O$\$ZnSnN_2$\Au solar cell, the statistics is based on 9 solar cells while the statistics is based on 8 solar cells for the Ag\ITO\$Cu_2O$\$ZnSnN_2$\Ag solar cell. The average V_{oc}, J_{sc}, FF and PCE of the solar cell with Au as the electrode are 0.22V, 1.75mA/cm^2, 42% and 0.15% while those of the solar cell with Ag as the electrode are 0.19V, 0.34mA/cm^2, 22% and 0.02%, respectively. Compared with the solar cell with Ag as the electrode, the solar cell with Au as the electrode has much larger J_{sc} due to smaller series resistance and this leads to larger fill factor and higher photoelectric conversion efficiency. After stored in air for 11 months without encapsulation, thought the performance degrades (the average V_{oc}, J_{sc}, FF and PCE of the solar cell with Au as the electrode are 0.06V, 0.56mA/cm^2, 27% and 0.01% after stored in air for 11 months), photovoltaic effect is still observed. The performance degradation might be due to the storage in air without encapsulation.

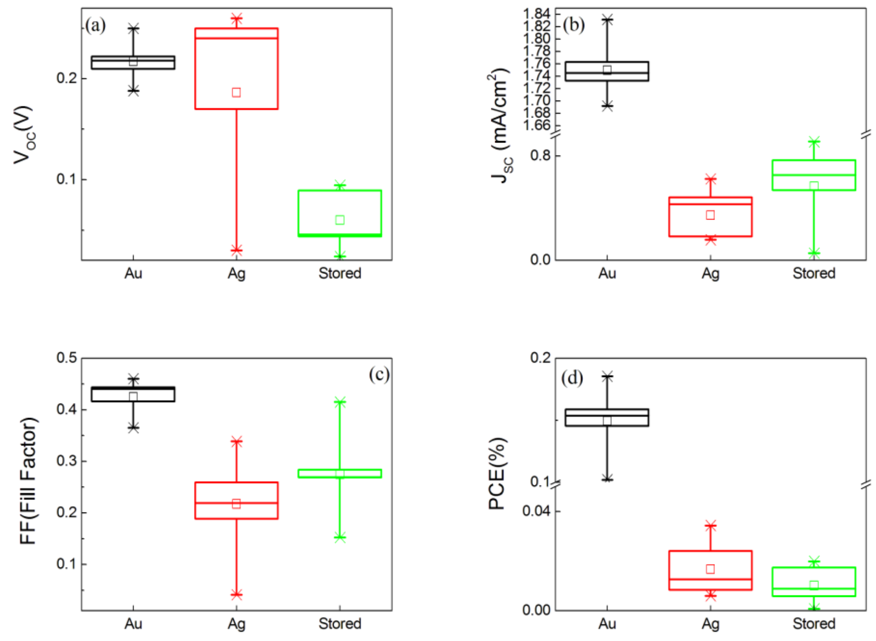

Figure S7. Statistical distribution of the performance factors for the solar cells: "Au" refers to the data from the Ag\ITO\$Cu_2O$\$ZnSnN_2$\Au solar cell without storage; "Ag" refers to the data from the Ag\ITO\$Cu_2O$\$ZnSnN_2$\Ag solar cell without storage; "Stored" refers to the data obtained after the Ag\ITO\$Cu_2O$\$ZnSnN_2$\Au solar cell has been stored in air without encapsulation for 11 months: (a) Open-circuit voltage V_{oc}; (b) Short-circuit current density J_{sc}; (c) FF(fill factor) and (d) PCE (power conversion efficiency).

The energy band gap diagram of Cu_2O-$ZnSnN_2$ is present in **Figure S8** [6,7]. The electron affinity of Cu_2O ($\chi(Cu_2O)$) is 3.1eV[6]. The bandgap of Cu_2O ($E_g(Cu_2O)$) is 2.1eV and the gap between E_F and E_v ($q\phi_p(Cu_2O)$) is 0.36eV[6]. The electron affinity of $ZnSnN_2$, χ, is 3.9eV. For $ZnSnN_2$, the gap between E_c and E_F, $q\phi_n$ is estimated to be 0.18eV ($E_c - E_F$ is calculated to be 0.18 eV from $n = N_c \exp[-(E_c - E_F)/kT]$ with n=10^{15}cm^{-3}, N_c=1.04×10^{18}cm^{-3}, kT=0.026eV). The band gap of $ZnSnN_2$, E_g, is 2.5 eV. V_D is the built-in potential between Cu_2O and $ZnSnN_2$. V_D is estimated to be 0.76 V from $\chi(Cu_2O)+E_g(Cu_2O)-q\phi_p(Cu_2O) = qV_D+\chi+q\phi_n$. The conduction band discontinuity ΔE_c is estimated to be 0.8 eV from $E_g(Cu_2O)-q\phi_p(Cu_2O) = qV_D+\Delta E_c+q\phi_n$. The valence band discontinuity ΔE_v is estimated to be 1.2 eV from $E_g-q\phi_n = q\phi_p(Cu_2O)+qV_D+\Delta E_v$.

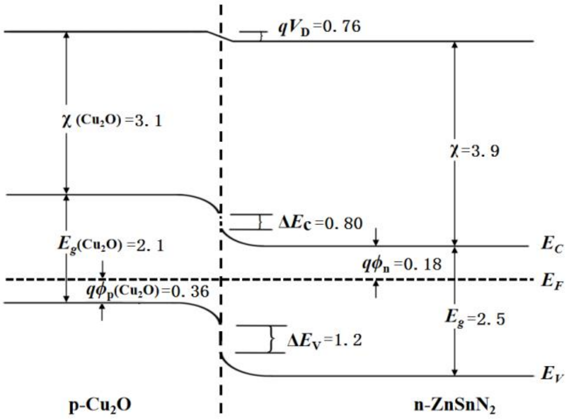

Figure S8. The energy band diagram of p-Cu_2O and n-$ZnSnN_2$ at zero bias.

Reference

[1] P. C. Quayle, K. He, J. Shan, K. Kash, Synthesis, lattice structure, and band gap of $ZnSnN_2$, MRS Commun. 3 (2013) 135-138.

[2] P. C. Quayle, G. T. Junno, K. He, E. W. Blanton, J. Shan, K. Kash, Vapour-liquid-solid synthesis of $ZnSnN_2$, Phys. Status Solidi B254 (2017) 1600718-1-1600718-6.

[3] F. Ye, Q.Q. Chen, X.M. Cai, Y.Z. Xie, X.F. Ma, K. Vaithinathan, D.P. Zhang, P. Fan, V.A.L. Roy, Improving the chemical potential of nitrogen to tune the electron density and mobility of $ZnSnN_2$, J. Mater. Chem. C 8 (2020) 4314-4320.

[4] P. C. Quayle, E. W. Blanton, A. Punya, G. T. Junno, K. He, L. Han, H. Zhao, J. Shan, W. R. L. Lambrecht, K. Kash, Charge-neutral disorder and polytypes in heterovalent wurtzite-based ternary semiconductors: The importance of the octet rule, Phys. Rev. B91 (2015) 205207-1-205207-14.

[5] S.M. Sze, K.K. Ng, Physics of semiconductor devices, 3rd edition(2006), Wiley Interscience, p.140.

[6] B. K. Meyer, A. Polity, D. Reppin, M. Becker, P. Hering, P. J. Klar, Th. Sander, C. Reindl, J. Benz, M. Eickhoff, C. Heiliger, M. Heinemann, J. Bl äsing, A. Krost, S. Shokovets, C. Müller, and C. Ronning, Binary copper oxide semiconductors: From materials towards devices, Phys. Status Solidi B 249(2012)1487–1509.

[7] K.K. Chinnakutti, V. Panneerselvam, S.T. Salammal, Tailoring optoelectronic properties of earth abundant $ZnSnN_2$ by combinatorial RF magnetron sputtering, J. Alloys Compd. 772 (2019) 348-358.
